# Editorial: New horizons in Alzheimer's disease research: combining cell, gene, and emerging therapies

**DOI:** 10.3389/fmed.2026.1919555

**Published:** 2026-07-20

**Authors:** Rebecca Piccarducci, Marco Mainardi, Laura Marchetti

**Affiliations:** 1 Department of Pharmacy, University of Pisa, Pisa, Italy; 2 Interdepartmental Center “Pisa Neuroscience” (PiNeuro), Pisa, Italy; 3 Department of Biomedical Sciences, University of Padua, Padua, Italy; 4 Padua Neuroscience Center, Padua, Italy

**Keywords:** Alzheimer's disease, amyloid-beta, extracellular vesicles (EVs), multi omics, neuroinflammation, precise medicine, synaptic dysfunction, tau pathology

Alzheimer's disease (AD) remains one of the most urgent challenges in modern biomedicine. Despite substantial advances over decades in defining its pathological hallmarks, i.e., amyloid-beta (Aβ) deposition and tau pathology (1), and other key processes involved in disease pathogenesis such as neuroinflammation (2) and synaptic dysfunction (3), effective disease-modifying therapies remain limited.

At the same time, the field has entered a more diversified phase, in which progresses are no longer driven by a single pathogenic hypothesis or therapeutic target. Instead, contemporary AD research increasingly brings together cellular mechanisms, molecular profiling, innovative disease models, and emerging interventions that extend beyond conventional pharmacology. The contributions in this Research Topic reflect this changing landscape and emphasize that further advances in AD will likely arise from the integration of such different research approaches.

A central theme emerging from this Research Topic is the need to refine the biological frameworks through which AD is studied. He et al. examine the intersection of genetic background and immune status as a basis for personalized immunotherapy in AD. By emphasizing the roles of APOE, which significantly contribute to develop a distinct genetic form of AD, immune biomarkers, and multi-omics integration, this review suggests that patient heterogeneity is not simply a clinical obstacle but an opportunity to design more precise and potentially more effective therapies. In particular, the review underscores how genetic risk and immune-state stratification may shape both disease trajectories and responsiveness to interventions, reinforcing the idea that precision medicine in AD will require more knowledge than amyloid or tau status alone. This perspective is especially timely as immunotherapeutic approaches continue to evolve and as the field seeks biomarkers capable of guiding patient selection and treatment monitoring (He et al.).

Importantly, progress toward biologically informed precision medicine also depends on the availability of experimental systems that can faithfully capture disease-relevant mechanisms.

In this context, Ghadami and Dellinger address a second major theme of the Topic: the need for improved models of tau-related pathology. Their study presents extracellular vesicle (EV)-mediated gene delivery as a biocompatible platform for generating tauopathy models *in vitro*. By showing that EVs can deliver tau-related plasmid cargo into Neuro-2a cells and that delivery parameters can be optimized for improved efficiency and reproducibility, this study contributes to develop more physiologically relevant and less disruptive tools for modeling disease processes. Such platforms are valuable not only for dissecting mechanisms of tau-mediated dysfunction, but also for enabling the screening and evaluation of candidate therapies in systems that more closely reflect biologically relevant modes of intercellular communication. As AD research continues to move toward earlier intervention and more targeted therapeutic design, robust and scalable models of tauopathy will remain indispensable (Ghadami and Dellinger). These advances in disease modeling are especially important because they create the experimental foundation for exploring therapeutic strategies that fall outside more conventional molecular approaches. Reflecting this broader translational perspective, Galeotti et al. investigate the use of low mechanical stimulation to counteract cytoskeletal and neuritic damage in cellular models of AD and related tauopathies. By demonstrating that mechanical stimulation can prevent axonal growth impairment and compensate for microtubule destabilization induced by tau- or amyloid-related stressors, the study points to the therapeutic relevance of neuronal biomechanics. These findings shift attention toward the physical stability of neuronal architecture as a modifiable component of neurodegeneration. In a field often dominated by biochemical targets, this work suggests that biomechanical interventions may represent an underappreciated yet potentially powerful avenue for neuroprotection. More broadly, it expands the conceptual framework for AD therapy by emphasizing that meaningful intervention may be achieved not only through targeting molecular pathology, but also through restoring the structural and functional integrity of neural systems (Galeotti et al.). This broader view of therapeutic intervention naturally extends from the preservation of neuronal structure at the cellular level to the modulation of brain activity at the systems level. This conceptual shift from cellular structure to network function is further developed by Zhao et al., who survey the physiological basis, therapeutic mechanisms, and translational prospects of light-based 40 Hz sensory stimulation for brain disorders, with particular emphasis on AD. Their review synthesizes evidence suggesting that gamma-frequency visual and audiovisual stimulation may entrain neural oscillations, modulate neuronal and glial activity, influence amyloid and tau pathology, and improve functional connectivity and cognitive outcomes. Importantly, it addresses the limitations and unresolved questions surrounding this approach, including mechanistic ambiguity, methodological heterogeneity, and challenges in clinical implementation. By doing so, the article captures both the promise of this strategy and the caution required in evaluating neuromodulatory interventions for AD. Its inclusion in the present Research Topic reflects the field's increasing willingness to consider therapies that act not only on molecular targets or cellular integrity, but also on circuit dynamics and systems physiology (Zhao et al.).

Taken together, the articles in this Research Topic underscore a fundamental shift in AD research toward more integrative approaches. They show that significant progress is emerging from the convergence of precision stratification, model innovation, mechanobiology, and neuromodulation. More specifically, they highlight the value of patient stratification informed by genetic and immune profiling, experimental systems capable of modeling disease processes with greater fidelity, and innovative interventions targeting not only molecular pathology, but also cellular architecture and network dynamics. Based on this vision, precision medicine, disease modeling, and emerging therapies are parts of the same translational framework rather than separate research fields. Such a perspective is especially important in a disorder as heterogeneous and multilevel as AD. The path forward will depend on our ability to link mechanistic understanding with clinically meaningful stratification, and therapeutic innovation with experimental rigor. The contributions assembled here provide valuable examples of this direction. Together, they suggest that the future of AD research lies in approaches capable of embracing, rather than simplifying, the biological complexity of the disease.
